# A Toxicity Prediction Tool for Potential Agonist/Antagonist Activities in Molecular Initiating Events Based on Chemical Structures

**DOI:** 10.3390/ijms21217853

**Published:** 2020-10-23

**Authors:** Kota Kurosaki, Raymond Wu, Yoshihiro Uesawa

**Affiliations:** Department of Medical Molecular Informatics, Meiji Pharmaceutical University, 2-522-1 Noshio, Kiyose, Tokyo 204-8588, Japan; d196955@std.my-pharm.ac.jp (K.K.); rwu.academic@gmail.com (R.W.)

**Keywords:** machine learning, nuclear receptor, stress response pathway, prediction model, molecular descriptor

## Abstract

Because the health effects of many compounds are unknown, regulatory toxicology must often rely on the development of quantitative structure–activity relationship (QSAR) models to efficiently discover molecular initiating events (MIEs) in the adverse-outcome pathway (AOP) framework. However, the QSAR models used in numerous toxicity prediction studies are publicly unavailable, and thus, they are challenging to use in practical applications. Approaches that simultaneously identify the various toxic responses induced by a compound are also scarce. The present study develops Toxicity Predictor, a web application tool that comprehensively identifies potential MIEs. Using various chemicals in the Toxicology in the 21st Century (Tox21) 10K library, we identified potential endocrine-disrupting chemicals (EDCs) using a machine-learning approach. Based on the optimized three-dimensional (3D) molecular structures and XGBoost algorithm, we established molecular descriptors for QSAR models. Their predictive performances and applicability domain were evaluated and applied to Toxicity Predictor. The prediction performance of the constructed models matched that of the top model in the Tox21 Data Challenge 2014. These advanced prediction results for MIEs are freely available on the Internet.

## 1. Introduction

Quantitative structure–activity relationship (QSAR) analysis is a technique used to predict the physiological activity of low-molecular-weight compounds based on their molecular structure [1,2]. In the field of toxicology, QSAR methodology is used for quantitative structure–toxicity relationship (QSTR) modeling using complex toxicity and adverse effect onset mechanisms that are objective variables [3,4].

An in silico approach, such as QSTR, is time and cost-effective for the detection of the potential toxicity of compounds in the early phases of drug development and pharmacovigilance, satisfying global ethical requirements regarding the 3R rules [5,6,7]. QSTR has therefore been extensively applied to regulatory toxicology. Recently, the critical application issue of realizing the implementation of toxicity prediction models extensively and of putting them to practical use has emerged. However, currently, one missing but desirable functionality in the practical use of QSTR prediction is that resources, such as the toxicity prediction models, should be distributed as highly convenient public software. Therefore, these toxicity prediction models should be published so that users can access QSTR prediction models for various toxicity targets [8,9,10].

The Toxicology in the 21st Century (Tox21) program is a consortium constituted by the National Institute of Health, the US Environmental Protection Agency, the National Toxicology Program, the National Center for Advancing Translational Sciences, and the Food and Drug Administration [11]. This project develops and evaluates novel efficient methods for toxicity assessments and mechanistic insights in addition to reducing time, costs, and animal usage [11,12]. Furthermore, in the ToxCast and Tox21 programs, for potentially molecular initiating event (MIE) targets for adverse outcome pathways [13,14], the in vitro quantitative high-throughput screening (qHTS) of approximately 10,000 compounds was performed [15]. These targets include nuclear receptors (NRs) and stress response (SR) pathways. Endocrine-disrupting chemicals (EDCs) interfere with the endocrine system by interacting with NRs and SR pathways and engender myriad adverse developmental, reproductive, neurological, and immunological effects in both humans and wildlife [16,17]. Therefore, identifying potential EDCs is of specific interest for the Tox21 program and environmental chemical hazard screening in general.

However, the in vitro qHTS assay is insufficient to screen all classes of chemicals, such as those still in the molecular development and optimization phase and, thus, cannot provide an accurate evaluation of the potential toxicity of chemicals in humans and the environment [18]. Therefore, a growing interest exists in a comprehensive in silico approach to detect the potential toxicity of chemicals. The literature presents the results of successful examples of alternative in silico toxicity screening methods and their applications using the Tox21 10K library [19,20,21]. However, even though there are 59 types of well-confirmed assay results of agonist/antagonist activities for toxicity targets in the Tox21 10K library, several studies have built models for only a small number of toxicity targets. There is still no comprehensive approach. Furthermore, because these models had not been opened, other researchers could not access the available constructed models. Therefore, users have found it challenging to perform and reuse the prediction of MIEs.

In this study, we overcame this problem by extensively collecting and processing databases of 59 types of assay targets based on the Tox21 10K library and constructed in silico toxicity prediction models for each assay target using XGBoost [22], which is a gradient-boosting algorithm with multiple uses for toxicity predictions [23]. The predictive performance of all models was validated and published on the web application. Using the prediction models constructed in this study, the screening of the potential toxicity of chemicals to various toxicity targets is possible.

## 2. Results and Discussion

### 2.1. Distributions of Active and Inactive Compounds

The PubChem activity scores were normalized between 0 and 100 using the following equation: activity = ((*V*_compound_ − *V*_DMSO_)/(*V*_pos_ − *V*_DMSO_)) × 100, where *V*_compound_, *V*_DMSO_, and *V*_pos_ denote the compound-well value, the median value of the DMSO-only wells, and the median value of the positive-control well, respectively [24]. The most active and inactive results have scores close to 100 and 0, respectively. In the PubChem documentation, all inactive compounds have a score of 0, active compounds have scores between 40 and 100, and inconclusive compounds have scores between 5 and 30. To implement the binary classification models, the binary teacher labels of active or inactive compounds were defined in two ways. In one definition, active compounds were scored 40 or higher; in another definition, active compounds were assigned scores of 1 or higher.

**Figure 1:**
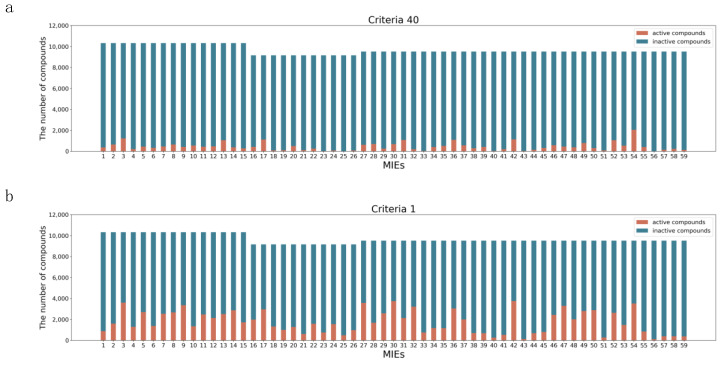
Activity distribution of 59 molecular initiating events (MIEs) in the Tox21 10K library: (**a**) the number of chemical compounds in the case of criteria 40 and (**b**) the number of chemical compounds in the case of criteria 1. Orange and blue show active and inactive chemicals, respectively.

We converted PubChem activity scores to binary labels using the two definitions of a criterion of 40 and a criterion of 1 to implement binary classification models for 59 toxicity targets. Figure 1a shows the number of active and negative compounds based on the definition of a criterion of 40, and Figure 1b shows that of a criterion of 1. For all toxicity targets, when we converted PubChem activity scores to binary labels with the definition of a criterion of 40, the mean ratio of active compounds to all compounds was 4.7% ± 4.0% and that of a criterion of 1 was 18.1% ± 11.0%. Lowering the criteria from 40 to 1 increased the mean ratio of active compounds by approximately 13.4%. However, when annotated with the criteria of 40, the ratio of active compounds in VDR_ago (PubChem activity score ID (AID): 743241), NFkB_ago (AID: 1159518), and TGFb_ago (AID: 1347035) were lower than 1%.

### 2.2. Models and Performances

**Figure 2:**
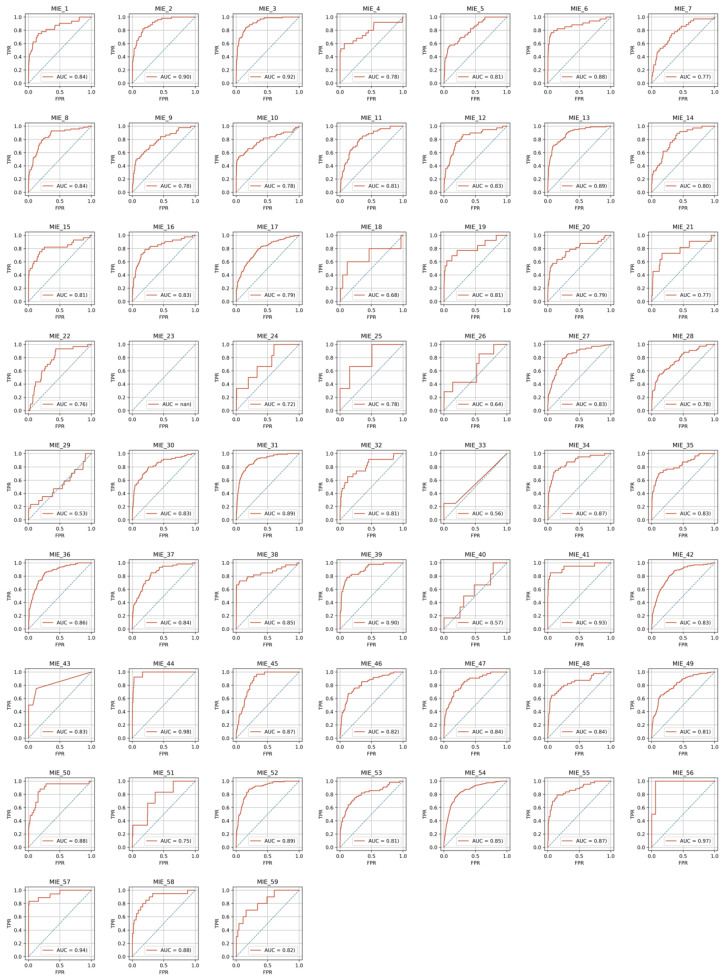
Receiver operating characteristic (ROC) curves with the test set in the case of criteria 40.

**Figure 3:**
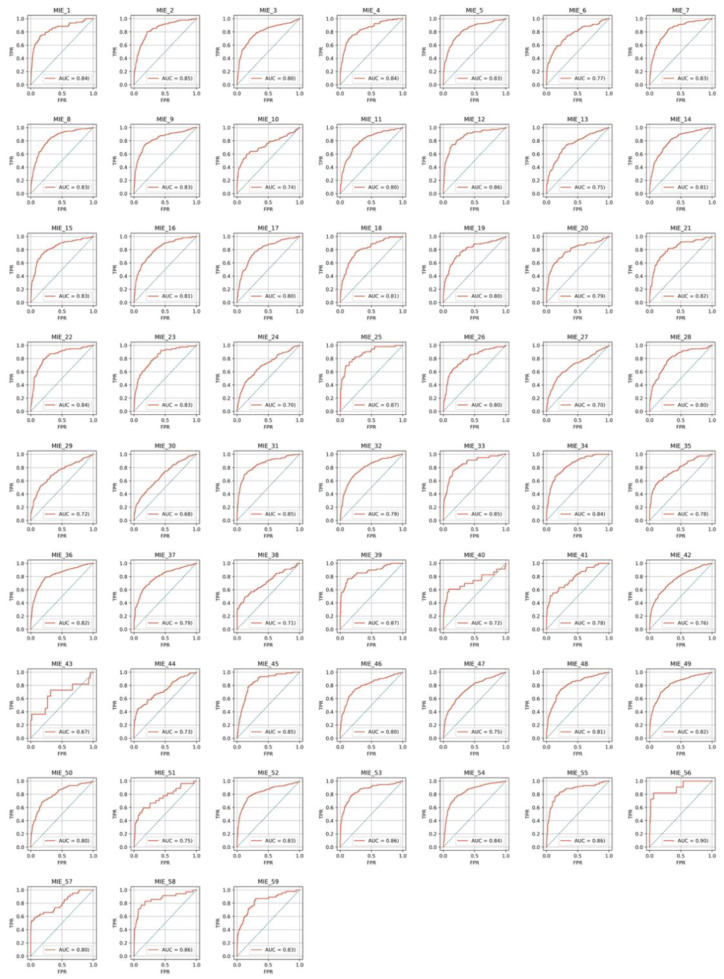
Receiver operating characteristic curves with the test set in the case of criteria 1.

For the 59 individual targets, 10% of all compounds was assigned to the test set. The other 90% of the compounds was used for the optimization, training, and validation of models in the validator, as shown in Section 3.5. The predictive performances of the constructed models were evaluated based on the area under the curve (AUC) of the receiver operating characteristic (ROC) curve in the test set. Optimal thresholds to convert the prediction probability to a binary class output were calculated using the Youden index gained from the ROC curve in the test set. Using these thresholds, predictive performances in the test set were evaluated. Table 1 shows the results of the test set. Model performances in the test set were evaluated using the metrics of AUC, sensitivity (SE), specificity (SP), accuracy (ACC), balanced accuracy (BAC), and the Matthews correlation coefficient (MCC). Figure 2 and Figure 3 show the ROC curves for all toxicity targets in the test set in the cases of criteria 40 and 1, respectively. In both cases in which the active labels were annotated with a criterion of 40 and 1, Table 2 summarizes the averages of predictive performances in the test set. Good predictive performances were observed for the models regardless of the criteria. However, for VDR_ago (AID: 743241), HIF1_ago (AID: 1224894), and Shh ago (AID: 1259390), which were annotated by a criterion of 40, the ratios of active compounds in the test set were 0%, 0.42%, and 0.62% and the AUC values were N.D., 0.556, and 0.571, respectively.

The classification performance of models tends to deteriorate because of class distribution imbalance [25]. A between-class imbalance degrades the prediction performance because of the bias in the prediction results toward the majority class, leading to more prediction errors in the minority class [26]. Figure 1 shows a sparser distribution of active compounds and an imbalance in the case of using a criterion of 40 compared to a criterion of 1. In this study, as shown in Figure 1 and Table 1, because of the between-class imbalance caused by the criteria of 40, constructing and evaluating some toxicity prediction models was impossible. We managed this problem by lowering the criteria from 40 to 1, and with this, we could evaluate the constructed models.

When using labels annotated by the criteria of 1, all compounds were treated as active, except those ensured to be inactive, which had a PubChem activity score of 0. Therefore, using the criterion of 1 in order to develop the models, we concluded that we developed criterion 1 models that accurately learned the inactive compounds compared with criterion 40 models. On the other hand, Judson et al. reported that a phenomenon called cytotoxicity-associated “burst” was observed for tests conducted on the Tox21 program [27]. Many chemicals show the activation of large numbers of assays over a narrow range of concentrations in which cell stress and cytotoxicity are also observed. Therefore, some of the assay activity in this concentration range may represent nonintentional chemical effects, such as cytotoxicity. Judson et al. [27] showed that the Tox21 10K library contains false positive responses induced by the burst phenomenon.

The quality of a machine learning model depends on that of the experimental data being fed into it. Ideally, machine learning models should be provided with reliable data for both active and inactive compounds during training; however, the current concern is that this decreases the number of active compounds being trained and increases the between-class imbalance in the data set being fed into the model. Consequently, the identification of burst compounds in our models has not yet been examined. Therefore, our models are still limited in terms of their ability to successfully feed the training data; particularly, their ability to exactly identify real active compounds remains a challenge. Importantly, the active compounds identified using our predictive models may actually be inactive. However, our models have learned nontoxic compounds more exactly than other approaches, and the ability to identify real negative compounds could be promising. A toxicity prediction model in the field of drug discovery must determine nontoxic compounds as well as must be capable of accurately determining toxic compounds; thus, our tool could practically aid in toxicity assessment application.

### 2.3. Comparison with the Tox21 Data Challenge 2014

For further validation of the predictive performance of the models established in this study, we compared their performance with the predictive models built in the Tox21 Data Challenge. The Tox21 Data Challenge 2014 was designed to understand the interference of the chemical compounds derived from the Tox21 10K library in the biological pathway using a crowd-sourced data analysis conducted by independent researchers. This challenge used data generated from seven NR and five SR signaling pathway assays to construct prediction models for QSARs [28].

There were 10 duplicate AIDs in the dataset used for in this challenge and in this study: AhR_ago (AID: 743122), Arlbd_ago (AID: 743053), ERlbd_ago (AID: 743077), Arom_ant (AID: 743139), PPAR-γ_ago (AID: 743140), ARE_ago (AID: 743219), ATAD5_ind (AID: 720516), HSR_act (AID: 743228), MMP_disr (AID: 720637), and p53_ago (AID: 720552). For construction of a model for each of these toxicity targets, the compounds used in this work overlapped with those used in the Tox21 Data Challenge. Moreover, the active and inactive compounds used in this work were defined using the annotation method based on the criteria of 40 and showed a 98.7% ± 0.7% match with the active and inactive compounds used in the challenge and showed strong concordance overall.

The allocations of the test sets used in the Tox21 Data Challenge were different from those used in this study. Therefore, a simple comparison using the predictive performance of the models used in the Tox21 Data Challenge and that constructed in this study is impossible. However, in this study, we established predictive models for the 10 duplicate toxicity targets using the equivalent compounds and teacher labels to those of the challenge. Therefore, the results of this challenge could be a performance benchmark to discuss the predictive performance of models built for the same targets in this study.

**Figure 4:**
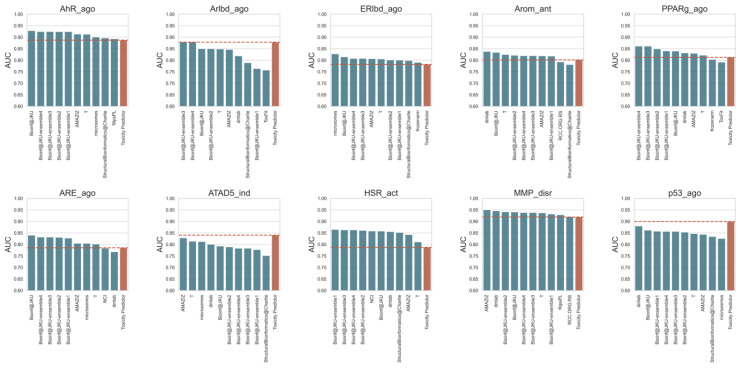
Comparison of the Toxicity Predictor models with the Tox21 Data Challenge 2014 models: This figure shows the predictive performance of the top 10 Tox21 Data Challenge and Toxicity Predictor models, which were built for 10 toxicity targets (AhR_ago, Arlbd_ago, ERlbd_ago, Arom_ant, PPARg_ago, ARE_ago, ATAD_ind, HSR_act, MMP_disr, and p53_ago). The horizontal axis denotes the names of the modeling teams of the Tox21 Data Challenge, and the vertical axis indicates the areas under the curve (AUCs).

The AUC has been adopted as a primary metric for ranking model performance in the Tox21 Data Challenge; therefore, the predictive models in the Tox21 Data Challenge have been ranked based on the AUC [29]. The AUCs in the test set validated in this study are shown in Figure 4. Although the predictive performances of models for four toxicity targets, i.e., models for AhR_ago (AID: 743122), ERlbd_ago (AID: 743077), MMP_disr (AID: 720637), and HSR_act (AID: 743228), achieved over an AUC of 0.750 and an accuracy score of over 0.846, their predictive performances were lower than that of the Tox21 Data Challenge models. On the other hand, six predictive models showed high AUCs: 0.878 (Arlbd_ago, AID: 743053), 0.801 (Arom_ant, AID: 743139), 0.813 (PPARg_ago, AID: 743140), 0.785 (ARE_ago, AID: 743219), 0.840 (ATAD5_ind, AID: 720516), and 0.899 (p53_ago, AID: 720552). The predictive performances for these six targets were comparable to or better than those of the top models of the Tox21 Data Challenge. Therefore, the results indicate that several predictive models developed in this study were valid toxicity models for in silico screening with high accuracy.

### 2.4. Implementation of the Models in the Toxicity Predictor

**Figure 5:**
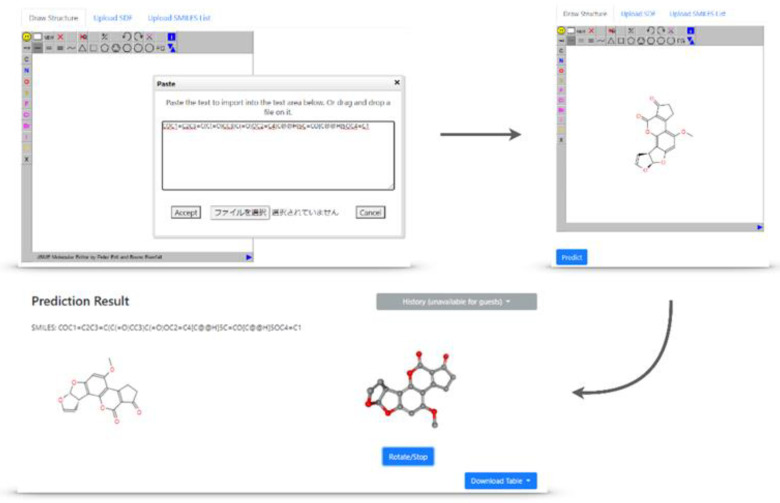
The platform screens of Toxicity Predictor.

**Figure 6:**
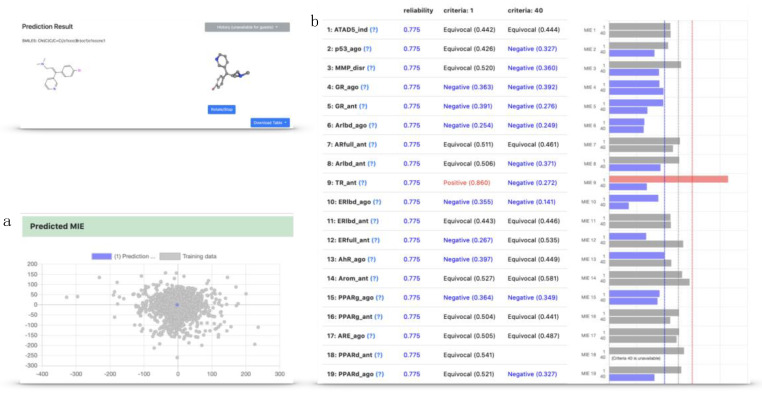
Prediction results in Toxicity Predictor: (**a**) the position of the compound to be predicted in the training set chemical space visualized with principal component analysis. The gray points are compounds in the training set, and the blue point is the compound to be predicted. (**b**) The predictive results for 59 MIEs predicted by Toxicity Predictor for each of the criteria 1 and 40. Normalized prediction scores for each target were displayed as bar charts. Red, blue, and gray bars show scores above 0.6, below 0.4, and between 0.4 and 0.6, respectively.

All 118 (two criteria for each of the 59 toxicity targets) models were implemented as part of Toxicity Predictor, which is a web application for the prediction of drug-induced liver injury. The Toxicity Predictor web application was constructed by the Development of a Drug Discovery Informatics System project in the Japan Agency for Medical Research and Development (AMED) and is available at http://mmi-03.my-pharm.ac.jp/tox1/. This application uses an input file containing one or multiple QSAR-ready structures in simplified molecular-input line-entry system (SMILES) strings or SDF format. Furthermore, it can depict a structural formula drawn in the browser and can use it as an input. The molecular structure from the input file is converted to a three-dimensional (3D) structure by the three-dimensionalization algorithm used in this study (Figure 5). Next, Toxicity Predictor calculated the necessary descriptors for the requested models using Mordred, an open-source software application used to calculate molecular descriptors. Finally, Toxicity Predictor predicted the chemical toxicity of 59 targets using the models constructed in this study. The prediction results of the input compound for the toxicity targets were converted to inactive or active, were returned, and can be viewed in a terminal browser (Figure 6b). Furthermore, the 3D structures and prediction results for all MIEs can be downloaded in SDF and CSV formats, respectively.

A model can be evaluated locally only within its applicability domain (AD), which is the chemical space of the training set [30,31]. Any extrapolation outside of that specific area of the structure space is most probably unreliable. Therefore, the system of the toxicity predictor incorporates domain evaluation to ensure the reliability of the QSTR inference. The AD of the evaluation compound is defined using the average of the logarithmic values of the Euclidean distance with the five nearest molecules in the descriptor space and is expressed numerically as reliability in Toxicity Predictor. Furthermore, the chemical structure is assessed to evaluate if it falls within the AD of the training set chemical space, and its position in the training set chemical space can be visualized and confirmed by principal component analysis (Figure 6a).

From the platform, entering the compounds for prediction and describing the chemical structure formula from an input format such as SMILES strings or SDF format is possible. The compound to be predicted is three-dimensionalized based on the algorithm in “Conformations and Descriptors”, and descriptors are calculated.

## 3. Materials and Methods

### 3.1. Biological Overview of Modeled MIEs

We outline the toxicological meanings of the endpoints established in our model construction research. The following cellular targets and their interactions with agonists and antagonists can be potential MIEs associated with diverse toxicological adverse outcomes (Tables S1 and S2).

AhR. The aryl hydrocarbon receptor (AhR), a member of the family of basic helix–loop–helix transcription factors, is crucial for the adaptation of responses to environmental changes. AhR is a ligand-activated transcription factor that is known to mediate most of the toxic and carcinogenic effects of various environmental contaminants such as polyaromatic hydrocarbons and dioxin [32].

GR. The glucocorticoid receptor (GR) is a member of the nuclear receptor family of ligand-dependent transcription factors. GR plays a critical role in carbohydrate, protein, and lipid metabolism and programmed cell death [33].

AR. The androgen receptor (AR), a nuclear hormone receptor, is significant in AR-dependent prostate cancer and other androgen-related diseases. EDCs and their interactions with steroid hormone receptors, such as AR, may disrupt normal endocrine function and interfere with metabolic homeostasis, reproduction, and developmental and behavioral functions [34].

ER and ERRs. The estrogen receptor (ER), a nuclear hormone receptor, plays an important role in development, metabolic homeostasis, and reproduction. Two subtypes of ER, ER-α and ER-β, are composed of various functional domains and have several structural regions in common [35]. EDCs and their interactions with steroid hormone receptors, such as ER, disrupt normal endocrine function. However, estrogen-related receptors (ERRs), the orphan nuclear receptors, are crucial in cellular energy metabolism control. ERR-α is a member of the NR superfamily, and studies have linked it with various cancers. In endocrine-related cancers, such as breast cancer, ERR-α regulates numerous target genes that direct cell proliferation and growth independent of ER-α [36].

PR. The progesterone receptor (PR), a nuclear hormone receptor, influences development, metabolic homeostasis, and reproduction. EDCs tend to bind to PR and disrupt normal endocrine function [37].

Aromatase. Aromatase catalyzes the conversion of androgen to estrogen and is vital in maintaining the androgen and estrogen balance in many EDC-sensitive organs [38].

TRHR. Thyrotropin-releasing hormone (TRH) receptor (TRHR) is a G-protein-coupled receptor (GPCR) that binds the tripeptide thyrotropin-releasing hormone. TRHR is found in the brain and, when bound by TRH, acts to increase the intracellular inositol trisphosphate through phospholipase C. It plays a crucial role in the anterior pituitary as it controls the synthesis and secretion of thyroid-stimulating hormone and prolactin [39].

TSHR. TSHR is a GPCR for thyrotropin (thyroid-stimulating hormone or TSH), which is a member of the glycoprotein hormone family. TSH is released by the anterior pituitary gland and is the main regulator of thyroid gland growth and development [40].

TR. Thyroid receptor (TR), a nuclear hormone receptor, plays an important role in normal brain development, metabolism control, and many aspects of normal adult physiology. A large number of industrial chemicals reduce circulating levels of thyroid hormone [41,42].

PPARs. Peroxisome proliferator-activated receptors (PPARs) are lipid-activated transcription factors of the NR superfamily with three distinct subtypes, namely PPAR-α, PPAR-δ (also called PPAR-β), and PPAR-γ. All these subtypes heterodimerize with Retinoid X receptor (RXR), and these heterodimers regulate the transcription of various genes. PPAR-γ receptor is involved in the regulation of glucose and lipid metabolism. The function of PPAR-δ includes the regulation of cholesterol and lipid metabolism [43].

FXR. Farnesoid X receptor (FXR), a member of the NR superfamily, is identified as a receptor of bile acids. It is found in large amounts in the liver, intestine, kidney, and adrenal cortex. FXR binds to FXR-response elements of DNA as a monomer or heterodimer with a common partner for NRs, RXR, to regulate the expression of the diverse genes involved in the metabolism of bile acids, lipids, and carbohydrates. Numerous studies have reported that FXR agonist is favorable for liver regeneration and hepatocarcinogenesis [44,45].

CAR. The constitutive androstane receptor (CAR) is a nuclear receptor that regulates gene expression for multiple drug-metabolizing enzymes and transporters, which are important factors in the metabolism of drugs or xenobiotics. CAR activation leads to the upregulation of organic anion transporting polypeptide (OATP) transporters—that is, hepatic uptake transporters—together with the upregulation of cytochrome P450 (CYP) and UDP-glucuronosyltransferases (UGT) enzymes [46].

PXR. Pregnane X receptor (PXR) regulates the expression of several drug-metabolizing enzymes, such as CYP3A4. The induction of these proteins is a major mechanism for developing drug resistance in cancer [47].

RAR. Retinoic acid receptor (RAR) is a nuclear receptor that regulates the development of chordate animals, including the body axis, spinal cord, forelimbs, heart, eye, and reproductive tract. Retinoic acid (RA) is derived from retinol (vitamin A) as a metabolic product and functions as a ligand for nuclear RARs. These RARs bind target genes as heterodimer complexes with RXRs at a DNA sequence known as the RA response element. Interference with RA signaling can have potential adverse effects on embryonic development [48].

ROR-γ. Nuclear receptor retinoic acid receptor-related orphan receptor gamma (ROR-γ) is a key transcription factor for the pathogenesis of autoimmune diseases mediated by Th17 cells. Because of the essential role of ROR-γ in controlling the differentiation and functioning of Th17 cells, interference with ROR-γ signaling pathways may promote susceptibility to immunotoxicants and autoimmune diseases.

RXRs. Retinoid X receptors (RXRs), with three distinct subtypes, namely RXR-α, RXR-β, and RXR-γ, occupy a central position in the NR superfamily, as they are common heterodimerization partners for several members of the human NRs, including PPARs, PXR, CAR, RARs, FXR, and TRs [49]. RXR-α has a potential role in metabolic signaling pathways, skin alopecia, dermal cysts, cardiac development, and insulin sensitization [50].

VDR. Vitamin D receptor (VDR), a member of the nuclear hormone receptor superfamily, plays a critical role in calcium homeostasis and bone metabolism [51].

ARE. The Nrf2–ARE pathway is an intrinsic mechanism of defense against oxidative stress. Nuclear factor E2-related factor 2 (Nrf2) is a transcription factor that induces the expression of target genes involved in the amelioration of oxidative stress by binding to the antioxidant response element (ARE) [52]. Oxidative stress can activate various transcription factors including NF-κB (nuclear factor-kappa B), AP-1 (activator protein-1), Nrf2, hypoxia-inducible factor-1 (HIF-1α), p53, and PPAR-γ. It can lead to chronic inflammation, mediating most chronic diseases, including cancer, diabetes, cardiovascular diseases, neurological diseases, and pulmonary diseases [53].

NF-κB and AP-1. The Nuclear factor-kappa B (NF-κB) transcription factor family and activator protein-1 (AP-1) transcription family are known as key regulators of inducible gene expression in the immune system [54].

HIF-1. Hypoxia-inducible factor-1 (HIF-1) is a major transcription factor that regulates the cellular response in low-oxygen conditions. HIF-1 comprises two subunits, hypoxia-responsive HIF-1-α and HIF-1-β, and is known as the aryl hydrocarbon receptor nuclear translocator. Under hypoxic conditions, HIF-1-α and HIF-1-β form a heterodimer. The HIF-1 complex translocates into the nucleus, binds to the hypoxia-responsive element (HRE), and activates the expression of target genes, such as vascular endothelial growth factor (VEGF). The HIF-1 pathway is essential for normal growth and development, and it is involved in the pathophysiology of cancer and inflammation [55].

p53. p53, a tumor suppressor protein, is activated following cellular insult, including DNA damage and other cellular stresses. The activation of p53 regulates cell fate by inducing DNA repair, cell cycle arrest, apoptosis, or cellular senescence. Therefore, the activation of p53 is a good indicator of DNA damage and other cellular stresses [56].

Casp. Caspases (Casps) involved in apoptosis are classified by their mechanism of action as initiator (caspase-2, -8, -9, and -10) and executioner caspases, classically described as the “executors of apoptosis” (caspase-3, -6, and -7). The inhibition of apoptosis results in numerous cancers, autoimmune diseases, inflammatory diseases, and viral infection [57].

HDAC. Histone deacetylases (HDACs) are a group of epigenetic enzymes that regulate gene expression by histone deacetylation. Histone acetylation plays a major and fundamental role in chromatin structure/function regulating eukaryotic gene expression, and it facilitates gene transcription and expression by relaxing the chromatin structure. HDAC inhibitors activate antitumor pathways through multiple action mechanisms, such as the activation of the apoptotic pathway and cell cycle arrest [58].

H2AX. One of the earliest cellular responses to DNA double-strand breaks is the phosphorylation at Ser139 of the core histone protein H2AX. This phospho-Ser139 serves as a sensitive biomarker for detecting such breaks, localizing the site of DNA repair [59].

HSR. Heat shock response (HSR) is a transcriptional response to elevated temperature shock, regulated by heat shock transcription factors (HSFs). The function of HSF-1, a well-studied target gene in HSR, is the protection of cells against proteotoxicity associated with misfolding, aggregation, and proteome mismanagement. While the induction of the HSR is specific to elevated temperature stress, a closely related cell stress response with HSF-1 is also induced when cells are exposed to other forms of environmental stress, such as oxidants, heavy metals, and xenobiotics, that cause protein damage and misfolding [60].

Shh. The hedgehog (Hh) pathway is crucial in many vital cellular processes, such as cell proliferation and differentiation during embryonic development. Three *Hh* genes discovered in vertebrates are Sonic Hedgehog (*Shh*), Indian Hedgehog (*Ihh*), and Desert Hedgehog (*Dhh*). Sonic Hedgehog protein (Shh) is the most widely found in adult tissues and is the most potent target. Therefore, chemicals that interfere with the Shh pathway are potential developmental toxicants [61].

TGF-β. Transforming growth factor-β (TGF-β) is a cytokine involved in various biological activities, including the regulation of proliferation, differentiation, and function of numerous cell types and the effects on glucose metabolism and fibrosis, in addition to its immunomodulatory function [62].

MMP. Mitochondrial membrane potential (MMP), a parameter for mitochondrial function, is generated by the mitochondrial electron transport chain that creates an electrochemical gradient. This gradient drives the synthesis of ATP, a crucial molecule for various cellular processes. Measuring MMP in living cells is commonly performed to assess the effect of chemicals on mitochondrial function [63].

ERsr. The endoplasmic reticulum (ER) plays a major role in the synthesis, folding, and structural maturation of proteins in the cell. If cells encounter conditions during which the workload imposed on the ER protein-folding machinery exceeds its capability, ER stress (ERsr) can occur. Under ERsr, secretory proteins start to accumulate in improperly modified and unfolded forms within the organelle [64].

ATAD5. Enhanced Level of Genome Instability Gene 1 (ELG1; human ATAD5) protein levels increase in response to various types of DNA damage. Thus, quantifying this activity can be used to identify the compounds that cause genetic stress [65].

### 3.2. Data Source

For this modeling study, data collection and processing work were conducted on the constructed toxic database based on Tox21. First, all datasets (training and test sets) of chemicals were downloaded in the SMILES format from the PubChem database, derived from the Tox21 program. We used a keyword for the database search, namely “Tox21 summary”, and selected bioassays of 59 toxicity targets, such as the NRs and SR pathways, to identify agonists/antagonists (Table 3). The toxicity scores (PubChem activity scores) of each toxic target were tied to the PubChem Substance IDs (SIDs). Finally, 14,250 compounds were used, but compounds with no activity score were excluded.

### 3.3. qHTS Data Analysis

**Figure 7:**
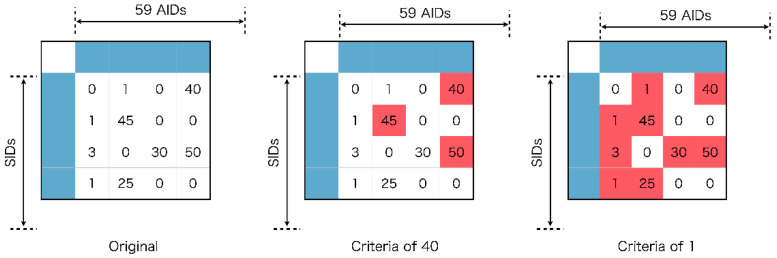
Relationship between the thresholds and active/inactive judgment. Red and white squares mean active and inactive judgments, respectively. Blue square means AIDs and SIDs.

The Tox21 10k library can rank the results of qHTS and prioritize hits according to PubChem activity scores. PubChem activity scores are assigned normalized scores between 0 and 100 for each PubChem activity score ID (AID). The most active results have scores closer to 100, and inactive scores are closer to 0. According to PubChem documentation, all inactive compounds have a score of 0, active compounds have scores between 40 and 100, and inconclusive compounds have scores between 5 and 30. In this study, to implement binary classification models, the binary labels of active or inactive compounds were adopted following two definitions: (1) Under the definition of a criterion of 40, compounds with scores from 40 to 100 were defined as active and those activity scores from 0 to 39 were defined as inactive. (2) Under the definition of a criterion of 1, compounds with scores from 1 to 100 were defined as active and those with activity scores of 0 were defined as inactive. In definition (1), only the compounds concluded to be active based on the Pubchem criterion were defined as active compounds, and the other compounds were defined as inactive even if they were inconclusive compounds. On the other hand, in definition (2), only the compounds concluded to be inactive based on the Pubchem criterion were defined as inactive compounds and the other compounds were treated as active compounds even if they were inconclusive compounds. In Figure 7, the scores highlighted in red show the active examples and other scores show inactive examples. Two types of binary label tables which denote active or inactive examples were created for the respective criteria.

The SIDs of the compounds used in this study are given in rows, and the AIDs are given in columns. The original table contains the original PubChem activity score of the compounds. In the table for the criteria of 40, the PubChem activity scores highlighted in red show the active examples for which the scores were larger than 40. In the table for the criteria of 1, the PubChem activity scores highlighted in red show active examples for which the scores were larger than 1.

### 3.4. Conformations and Descriptors

SMILES strings were cleaned and standardized (removing salts, counterions, and fragments and adjusting the protonation state (neutralize)) by RDkit, which is a Python library [66]. Optimal 3D structures were generated by following a calculation process to handle the calculation of excessive candidate compounds using an efficient and heuristic—though not strictly ideal—method. First, chemical structures were generated from the SMILES strings, and explicit hydrogen atoms were added to the chemical structures. Next, up to 200 types of 3D conformers were randomly generated. The energy minimization calculation was performed on them by the MMFF force field, and a conformer with minimal energy was adopted from 200 types of conformers. However, when this process lasted more than 60 s, instead of the above calculations, the conformer was generated using the ETKDG method [67] and the energy minimization calculation was performed on it by the MMFF force field [68]. Finally, the optimal conformer was converted into an SDF format.

Molecular descriptors were calculated for each compound using Mordred [69,70], a Python library; 2D and 3D descriptors were obtained; and finally, 1824 descriptors were adopted for model construction.

### 3.5. ML Algorithm and Modeling Scheme

**Figure 8:**
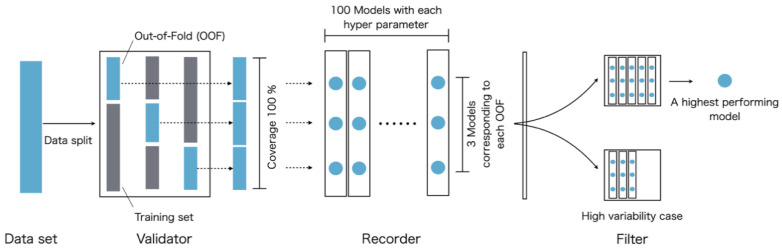
The modeling pipeline integrated validator, recorder, and filter used in this study.

Classification models based on Tox21 were developed using XGBoost. This algorithm was designed to be highly scalable by adopting a sparsity-aware algorithm for sparse data and a weighted quantile sketch for approximate tree learning [22]. In this study, the modeling scheme was designed to integrate the validator, recorder, and filter to gain a single model satisfying high-predictive performance and robustness (Figure 8). Further, 10% of all compounds was assigned to the test set without the data being fed into this pipeline. The compounds fed into the pipeline included 90% of all compounds obtained by excluding the test set, and these were used for the optimization, training, and validation of the models.

Validator. In the validator, hyperparameter exploration using a grid search was performed. ML models were trained and validated according to the respective grid-generated parameter values. One-third of the data fed into this validator was assigned to the validation set as out-of-fold (OOF) and two-thirds to the training set, where the predictive performance was validated using the hold-out method. Here, when assigning validation and training sets, extreme unlike distributions between the validation and training sets could occur by chance. Therefore, three patterns of allocations of OOF were generated, ensuring that it represented 100% coverage of the input data set and without duplication. For all pairs of validation training set allocations, the models were constructed using each grid-generated hyperparameter. They evaluated the predictive performance in the validation sets according to the ROC-AUC. The hyperparameter governing the performance of the XGBoost was explored within the following predefined ranges: learning rate (“learning_rate”: 100 types of values from 0.01 × 0 to 0.01 × 99).

Recorder. The recorder works as a record-keeper for the validator. The number of conditions to evaluate in the validator reached 300 patterns consisting of three OOFs and 100 hyperparameters. This recorder stored all prediction models constructed for the respective conditions, their modeling conditions, and the predictive performances in the OOFs.

Filter. The filter eliminates some overfitting cases while selects the models with the highest predictive performance from the information stored in the recorder. In the filter, based on 300 prediction performances stored in the recorder, a set of the highest predictive performing models and their modeling conditions was selected. Here, we imposed the following request to detect some overfitting cases. We excluded some hyperparameters used for model construction when the models with this hyperparameter had a high variability of the predictive performances between other OOFs in the 100% coverage validation. Therefore, even if the selected set of hyperparameters and allocation of OOFs resulted in high predictive performance, it was not adopted when the variability of performance with other OOFs at a coverage of 100% was high.

In the validator, using three types of unduplicated out-of-folds (OOFs) as the validation set, models were trained and validated with each hyperparameter. In the recorder, all prediction models, their modeling conditions, and predictive performances were stored. In the filter, high-variability cases were excluded according to 100% coverage validation, and the highest performing model was selected simultaneously.

### 3.6. Evaluation Metrics

The predictive performance of the classification models was evaluated based on information calculated from confusion matrices, including the number of true positives (*TP*; compounds correctly identified as positive), true negatives (*TN*; compounds correctly identified as negative), false negatives (*FN*; misclassified positive compounds), and false positives (*FP*; misclassified negative compounds). The following six evaluation indexes were used to evaluate the classification models.

(1) *SE*: accuracy of predicting “positive” (active) when the true outcome is positive.

(1) $$SE=\frac{TP}{TP+FN}$$

(2) *SP*: accuracy of predicting “negative” (inactive) when the true outcome is negative.

(2) $$SP=\frac{TN}{TN+FN}$$

(3) *ACC*: the number of correctly predicted samples divided by the total number of samples.

(3) $$ACC=\frac{TP+TN}{TP+TN+FN+FP}$$

(4) *BAC*: average between *SE* and *SP*.

(4) $$BAC=\frac{1}{2}\left(SE+SP\right)$$

(5) *MCC*: used as a measure to assess the classification accuracy of the models for an unbalanced dataset [71].

(5) $$MCC=\frac{\left(TP\cdot TN\right)-\left(FP\cdot FN\right)}{\sqrt{\left(TP+FP\right)\left(TP+FN\right)\left(TN+FP\right)\left(TN+FN\right)}}$$

(6) *AUC*: a graph showing the performance of a classification model at all classification thresholds. This curve plots two parameters: (i) *SE* and (ii) 1–*SP* [72].

To determine the optimal cutoff points in the definitions of *TP*, *FN*, *TN*, and *FP*, we maximized *SE* (1–*SP*) using the Youden index [73]. In the toxicity predictor, the cutoff value specific to each prediction model was standardized and displayed using the following formula so that the maximum, minimum, and average values were 1, 0, and 0.5, respectively.

(6) $$x_{n}=\;x_{u}^{-\log_{c}2}$$

The value *x_n_* is obtained by normalizing the directly predicted value *x_u_* using the equation. Here, *c* is the cutoff value of each prediction model.

### 3.7. Applicability Domain

The AD of the compound entered for the prediction was defined using the Euclidean distance to the five nearest molecules in the descriptor space of Tox21 compounds. The mean of the logarithmic Euclidean distances was normalized between 0 and 1 and expressed as reliability in the toxicity predictor.

## 4. Conclusions

In this study, we built prediction models of 59 MIE agonists and antagonists with information on the chemical structure and activity from the Tox21 10K library. We aimed to support regulatory toxicity decisions comprehensively and to enable users to reuse the QSTR predictions. Therefore, a web application integrating the three-dimensionalization algorithm, toxicity prediction models, and domain evaluation used in this study was developed to access to the assessment of activity against 59 MIEs. These models were valid toxicity models for alternative in silico screening and therefore could practically aid in achieving toxicity assessment.

## Figures and Tables

**Table 1:** Predictive performances in the test set for each target.

No.	AID	Abbreviation	Criteria 40						Criteria 1					
			AUC	SE	SP	ACC	BAC	MCC	AUC	SE	SP	ACC	BAC	MCC
1	720516	ATAD5_ind	0.840	0.750	0.843	0.840	0.796	0.272	0.845	0.744	0.847	0.839	0.795	0.395
2	720552	p53_ago	0.899	0.824	0.830	0.830	0.827	0.356	0.845	0.804	0.793	0.794	0.799	0.458
3	720637	MMP_disr	0.919	0.845	0.846	0.846	0.845	0.501	0.795	0.698	0.788	0.758	0.743	0.475
4	720719	GR_ago	0.783	0.600	0.931	0.923	0.766	0.300	0.841	0.754	0.807	0.800	0.780	0.416
5	720725	GR_ant	0.808	0.577	0.905	0.888	0.741	0.328	0.827	0.801	0.721	0.743	0.761	0.471
6	743053	Arlbd_ago	0.878	0.765	0.947	0.941	0.856	0.481	0.766	0.582	0.843	0.806	0.712	0.357
7	743054	ARfull_ant	0.774	0.750	0.681	0.683	0.716	0.169	0.833	0.841	0.700	0.734	0.770	0.468
8	743063	Arlbd_ant	0.844	0.786	0.791	0.790	0.788	0.338	0.833	0.805	0.724	0.745	0.765	0.469
9	743067	TR_ant	0.783	0.511	0.924	0.906	0.718	0.306	0.829	0.740	0.825	0.796	0.782	0.555
10	743077	ERlbd_ago	0.782	0.536	0.961	0.938	0.748	0.457	0.735	0.600	0.843	0.812	0.722	0.362
11	743078	ERlbd_ant	0.810	0.815	0.684	0.691	0.750	0.237	0.805	0.696	0.789	0.767	0.743	0.444
12	743091	ERfull_ant	0.826	0.872	0.699	0.705	0.785	0.235	0.862	0.730	0.870	0.842	0.800	0.555
13	743122	AhR_ago	0.888	0.713	0.907	0.887	0.810	0.513	0.749	0.728	0.695	0.702	0.711	0.359
14	743139	Arom_ant	0.801	0.892	0.598	0.608	0.745	0.186	0.807	0.825	0.661	0.704	0.743	0.429
15	743140	PPARg_ago	0.813	0.750	0.823	0.821	0.786	0.238	0.832	0.735	0.819	0.805	0.777	0.457
16	743199	PPARg_ant	0.829	0.786	0.798	0.798	0.792	0.290	0.810	0.824	0.645	0.682	0.734	0.383
17	743219	ARE_ago	0.785	0.794	0.652	0.672	0.723	0.317	0.795	0.770	0.715	0.733	0.742	0.461
18	743226	PPARd_ant	0.681	0.600	0.885	0.884	0.743	0.111	0.811	0.764	0.749	0.751	0.756	0.374
19	743227	PPARd_ago	0.812	0.615	0.954	0.949	0.785	0.296	0.796	0.705	0.790	0.780	0.747	0.356
20	743228	HSR_act	0.788	0.576	0.922	0.910	0.749	0.315	0.790	0.667	0.808	0.789	0.737	0.370
21	743239	FXR_ago	0.775	0.727	0.836	0.835	0.782	0.163	0.817	0.689	0.834	0.825	0.762	0.325
22	743240	FXR_ant	0.757	0.933	0.565	0.577	0.749	0.178	0.843	0.788	0.799	0.798	0.794	0.481
23	743241	VDR_ago	N.D	N.D	N.D	N.D	N.D	N.D	0.826	0.769	0.727	0.730	0.748	0.297
24	743242	VDR_ant	0.716	1.000	0.399	0.403	0.699	0.066	0.701	0.630	0.689	0.678	0.660	0.258
25	1159518	NFkB_ago	0.780	0.667	0.846	0.846	0.756	0.081	0.871	0.692	0.912	0.900	0.802	0.427
26	1159519	ERsr_ago	0.638	0.857	0.441	0.445	0.649	0.052	0.801	0.655	0.833	0.816	0.744	0.349
27	1159523	ROR_ant	0.828	0.789	0.764	0.766	0.777	0.323	0.695	0.523	0.819	0.703	0.671	0.359
28	1159528	AP1_ago	0.777	0.553	0.877	0.851	0.715	0.319	0.799	0.765	0.722	0.729	0.743	0.372
29	1159531	RXR_ago	0.532	0.235	0.964	0.951	0.600	0.135	0.725	0.527	0.841	0.756	0.684	0.374
30	1159555	RAR_ant	0.831	0.800	0.742	0.746	0.771	0.308	0.683	0.740	0.511	0.601	0.626	0.249
31	1224892	CAR_ago	0.889	0.826	0.808	0.810	0.817	0.455	0.847	0.684	0.889	0.845	0.787	0.556
32	1224893	CAR_ant	0.809	0.652	0.880	0.874	0.766	0.239	0.793	0.700	0.768	0.746	0.734	0.448
33	1224894	HIF1_ago	0.556	0.250	1.000	0.997	0.625	0.499	0.854	0.769	0.829	0.824	0.799	0.395
34	1224895	TSHR_ago	0.872	0.750	0.880	0.874	0.815	0.355	0.838	0.692	0.831	0.816	0.762	0.389
35	1224896	H2AX_ago	0.834	0.696	0.892	0.880	0.794	0.394	0.779	0.605	0.842	0.814	0.724	0.354
36	1259247	Arfulls_ant	0.856	0.857	0.733	0.747	0.795	0.401	0.824	0.788	0.767	0.774	0.778	0.534
37	1259248	Erfulls_ant	0.835	0.850	0.702	0.711	0.776	0.283	0.793	0.668	0.798	0.770	0.733	0.416
38	1259387	ARant_ago	0.852	0.727	0.946	0.939	0.837	0.460	0.712	0.494	0.872	0.841	0.683	0.275
39	1259388	HDAC_ant	0.897	0.783	0.888	0.883	0.835	0.407	0.868	0.768	0.879	0.871	0.824	0.447
40	1259390	Shh_ago	0.571	1.000	0.219	0.223	0.609	0.042	0.724	0.609	0.913	0.905	0.761	0.266
41	1259391	ERaant_ago	0.934	0.850	0.959	0.956	0.904	0.493	0.782	0.551	0.898	0.880	0.725	0.299
42	1259392	Shh_ant	0.829	0.809	0.718	0.731	0.764	0.379	0.758	0.642	0.745	0.705	0.693	0.383
43	1259393	TSHR_agoant	0.834	0.750	0.875	0.874	0.812	0.120	0.669	0.727	0.681	0.682	0.704	0.093
44	1259394	ERb_ago	0.980	0.923	0.973	0.972	0.948	0.531	0.729	0.444	0.937	0.900	0.691	0.348
45	1259395	TSHR_ant	0.865	0.933	0.715	0.721	0.824	0.244	0.850	0.800	0.807	0.807	0.804	0.381
46	1259396	Erb_ant	0.825	0.677	0.863	0.851	0.770	0.352	0.798	0.743	0.763	0.758	0.753	0.462
47	1259401	ERRPGC_ant	0.843	0.698	0.843	0.837	0.770	0.290	0.751	0.595	0.793	0.723	0.694	0.390
48	1259402	ERRPGC_ago	0.840	0.650	0.937	0.925	0.794	0.415	0.805	0.734	0.777	0.768	0.756	0.444
49	1259403	ERR_ant	0.812	0.653	0.856	0.835	0.755	0.392	0.819	0.696	0.826	0.786	0.761	0.510
50	1259404	ERR_ago	0.884	0.880	0.814	0.816	0.847	0.274	0.803	0.680	0.820	0.777	0.750	0.491
51	1347030	TRHR_ago	0.748	0.833	0.637	0.638	0.735	0.077	0.751	0.593	0.853	0.846	0.723	0.201
52	1347031	PR_ant	0.892	0.880	0.794	0.804	0.837	0.473	0.831	0.757	0.821	0.802	0.789	0.550
53	1347032	TGFb_ant	0.809	0.750	0.765	0.764	0.757	0.273	0.860	0.780	0.824	0.817	0.802	0.493
54	1347033	PXR_ago	0.851	0.759	0.817	0.805	0.788	0.517	0.838	0.745	0.817	0.790	0.781	0.556
55	1347034	CaspH_ind	0.870	0.791	0.852	0.849	0.821	0.348	0.858	0.773	0.856	0.848	0.814	0.452
56	1347035	TGFb_ago	0.968	1.000	0.938	0.938	0.969	0.174	0.900	0.818	0.937	0.936	0.878	0.311
57	1347036	PR_ago	0.943	0.833	0.989	0.986	0.911	0.701	0.799	0.537	0.986	0.967	0.761	0.564
58	1347037	CaspC_ind	0.884	0.850	0.785	0.786	0.817	0.216	0.863	0.771	0.882	0.878	0.827	0.351
59	1347038	TRHR_ant	0.822	0.700	0.841	0.840	0.771	0.148	0.828	0.870	0.701	0.709	0.785	0.260

AID means PubChem assay IDs. Predictive performances were evaluated using the following metrics: area under the curve of receiver operating characteristic curve (AUC), sensitivity (SE), specificity (SP), accuracy (ACC), balanced accuracy (BAC), and Matthews correlation coefficient (MCC). N.D. shows no data.

**Table 2:** Mean predictive performances for all assay targets.

Metrics	Criteria 40	Criteria 1
AUC	0.817 ± 0.088	0.802 ± 0.051
SE	0.750 ± 0.151	0.705 ± 0.094
SP	0.809 ± 0.149	0.801 ± 0.082
ACC	0.807 ± 0.144	0.788 ± 0.069
BAC	0.780 ± 0.069	0.753 ± 0.045
MCC	0.307 ± 0.141	0.402 ± 0.096

Each value of performances evaluated by six metrics were shown as mean ± standard error. *n* = 58 (criteria 40), *n* = 59 (criteria 1).

**Table 3:** Molecular Initiating Events (MIEs) used in this study.

No.	AID	Molecular Initiating Events	Activity Type	Abbreviation
1	720516	ATAD5	genotoxic inducer	ATAD5_ind
2	720552	p53	agonist	p53_ago
3	720637	mitochondrial membrane potential	disruptor	MMP_disr
4	720719	glucocorticoid receptor	agonist	GR_ago
5	720725	glucocorticoid receptor	antagonist	GR_ant
6	743053	androgen receptor lbd	agonist	Arlbd_ago
7	743054	androgen receptor full	antagonist	ARfull_ant
8	743063	androgen receptor lbd	antagonist	Arlbd_ant
9	743067	thyroid receptor	antagonist	TR_ant
10	743077	estrogen receptor alpha lbd	agonist	ERlbd_ago
11	743078	estrogen receptor alpha lbd	antagonist	ERlbd_ant
12	743091	estrogen receptor alpha full	antagonist	ERfull_ant
13	743122	aryl hydrocarbon receptor	agonist	AhR_ago
14	743139	aromatase	antagonist	Arom_ant
15	743140	peroxisome proliferator-activated receptor gamma	agonist	PPARg_ago
16	743199	peroxisome proliferator-activated receptor gamma	antagonist	PPARg_ant
17	743219	antioxidant response element	agonist	ARE_ago
18	743226	peroxisome proliferator-activated receptor delta	antagonist	PPARd_ant
19	743227	peroxisome proliferator-activated receptor delta	agonist	PPARd_ago
20	743228	heat shock response	activator	HSR_act
21	743239	farnesoid-X-receptor	agonist	FXR_ago
22	743240	farnesoid-X-receptor	antagonist	FXR_ant
23	743241	vitamin D receptor	agonist	VDR_ago
24	743242	vitamin D receptor	antagonist	VDR_ant
25	1159518	NFkB	agonist	NFkB_ago
26	1159519	endoplasmic reticulum stress response	agonist	ERsr_ago
27	1159523	retinoid-related orphan receptor gamma	antagonist	ROR_ant
28	1159528	activator protein-1	agonist	AP1_ago
29	1159531	retinoid X receptor-alpha	agonist	RXR_ago
30	1159555	retinoic acid receptor	antagonist	RAR_ant
31	1224892	constitutive androstane receptor	agonist	CAR_ago
32	1224893	constitutive androstane receptor	antagonist	CAR_ant
33	1224894	hypoxia	agonist	HIF1_ago
34	1224895	thyroid stimulating hormone receptor	agonist	TSHR_ago
35	1224896	histone variant H2AX	agonist	H2AX_ago
36	1259247	androgen receptor with stimulator	antagonist	Arfulls_ant
37	1259248	estrogen receptor alpha with stimulator	antagonist	Erfulls_ant
38	1259387	androgen receptor with antagonist	agonist	ARant_ago
39	1259388	histone deacetylase	antagonist	HDAC_ant
40	1259390	sonic hedgehog signaling	agonist	Shh_ago
41	1259391	estrogen receptor alpha with antagonist	agonist	ERaant_ago
42	1259392	sonic hedgehog signaling	antagonist	Shh_ant
43	1259393	thyroid stimulating hormone receptor	agonist antagonist	TSHR_agoant
44	1259394	estrogen receptor beta	agonist	ERb_ago
45	1259395	thyroid stimulating hormone receptor	antagonist	TSHR_ant
46	1259396	estrogen receptor beta	antagonist	Erb_ant
47	1259401	estrogen related receptor with PGC	antagonist	ERRPGC_ant
48	1259402	estrogen related receptor with PGC	agonist	ERRPGC_ago
49	1259403	estrogen related receptor	antagonist	ERR_ant
50	1259404	estrogen related receptor	agonist	ERR_ago
51	1347030	thyrotropin releasing hormone receptor	agonist	TRHR_ago
52	1347031	progesterone receptor	antagonist	PR_ant
53	1347032	transforming growth factor beta	antagonist	TGFb_ant
54	1347033	human pregnane X receptor	agonist	PXR_ago
55	1347034	caspase-3/7 in HepG2	inducer	CaspH_ind
56	1347035	transforming growth factor beta	agonist	TGFb_ago
57	1347036	progesterone receptor	agonist	PR_ago
58	1347037	caspase-3/7 in CHO-K1	inducer	CaspC_ind
59	1347038	thyrotropin releasing hormone receptor	antagonist	TRHR_ant

AID means PubChem assay IDs.

## Supplementary Materials

Supplementary Materials can be found at https://www.mdpi.com/1422-0067/21/21/7853/s1.
